# Supplementary material for: Deep learning with photonic neural cellular automata
Source: Light Sci Appl. 2024 Oct 8;13:283. doi: 10.1038/s41377-024-01651-7 (PMC11461964; doi:10.1038/s41377-024-01651-7)
Supplement: Supplementary file 1 — Supplementary Information [file 41377_2024_1651_MOESM1_ESM.pdf]

Supplementary Information for Deep Learning with Photonic Neural Cellular Automata

Gordon H.Y. Li,[1] Christian R. Leefmans,[1] James Williams,[2]
Robert M. Gray,[2] Midya Parto,[2,3] and Alireza Marandi[1,2,*]

[1] *Department of Applied Physics, California Institute of Technology, Pasadena, CA 91125, USA*

[2] *Department of Electrical Engineering, California Institute of Technology, Pasadena, CA 91125, USA*

[3] *Physics and Informatics Laboratories, NTT Research, Inc., Sunnyvale, California 94085, USA*

[*] marandi@caltech.edu

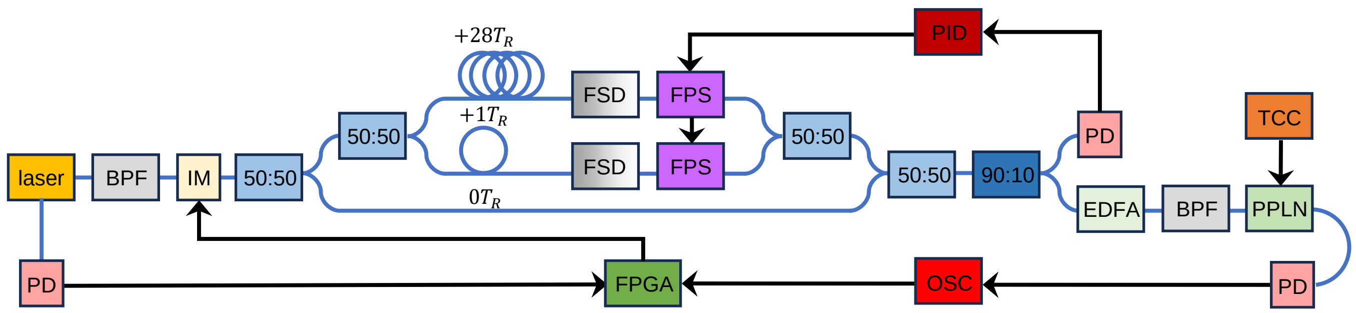

FIG. S1. **Detailed schematic of experimental setup.** BPF: band-pass filter, IM: intensity modulator, FSD: free-space delay stage, FPS: fiber phase-shifter, EDFA: erbium-doped fiber amplifier, PD: photodetector, PPLN: periodically-poled lithium niobate, TCC: thermocouple controller, PID: proportional integral derivative controller, OSC: oscilloscope, FPGA: field programmable gate array. Blue lines represent single-mode polarization-maintaining optical fiber paths and black lines represent electronic connections.

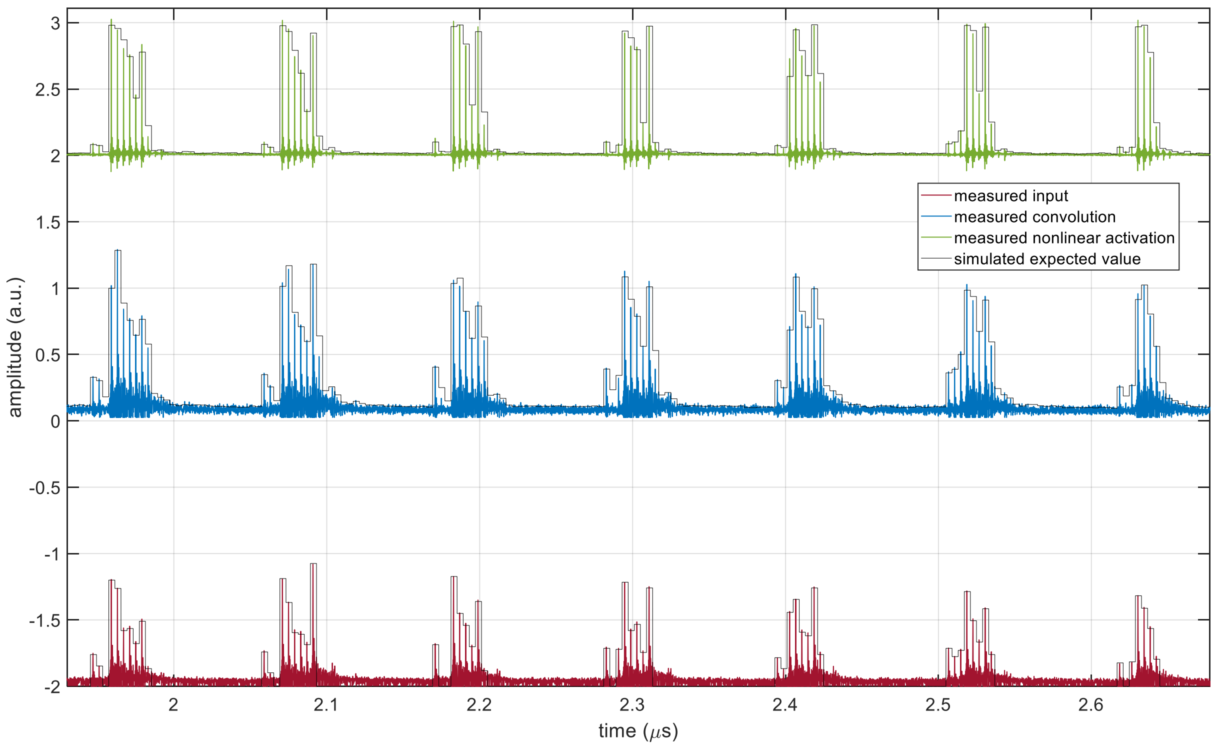

FIG. S2. **Example of experimentally-obtained time traces in photonic neural cellular automata.** Oscilloscope time traces for a portion of one iteration of one image in the photonic neural cellular automata showing the results of the input cell states (red line), linear convolution (blue line), and nonlinear activation (green line) compared against the expected values based on digital simulations (black lines). The peak or maximum amplitudes of each light pulse, which occupy a specific time bin in the synthetic temporal lattice, agrees well with the expected cell state values and shows that the calibration was accurate. The time traces for each operation are shifted vertically and corresponding cell lattice sites are aligned horizontally in time for clarity.

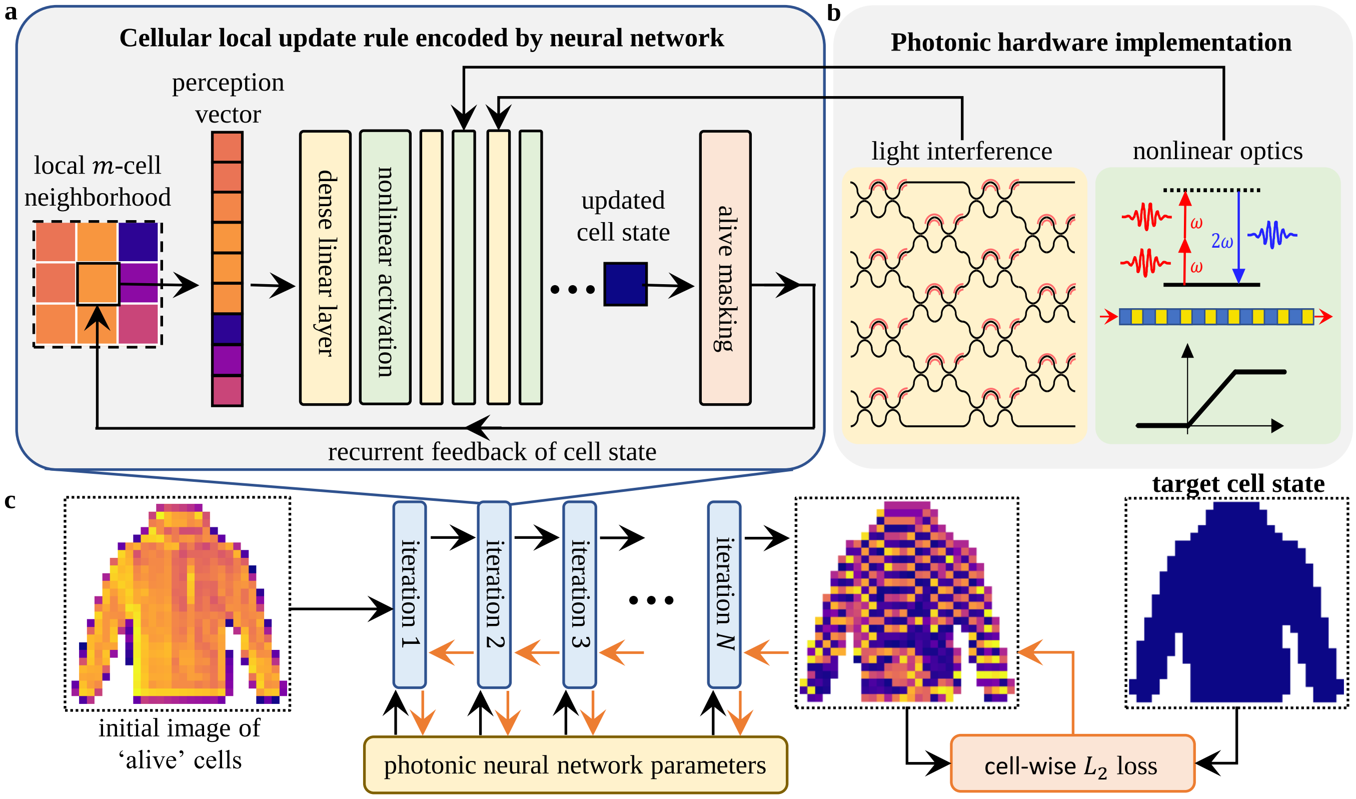

FIG. S3. **Training method for photonic neural cellular automata.** (a) A single iteration of PNCA consists of alive cells that are encoded into an optical signal, m optical paths encoding a local m-cell neighborhood and perception vector for each cell, updating the state of each cell according to a local update rule represented by a neural network, and alive cell masking. (b) Photonic hardware encodes the local update rule, which includes linear operations implemented physically via light interference, and nonlinear operations implemented physically via nonlinear optics. (c) Backpropagation-through-time algorithm for training PNCA to learn a local update rule, which upon repeated iteration causes self-organization of cells for an image classification task. A cell-wise L_2 loss is used for optimizing the photonic neural network parameters.

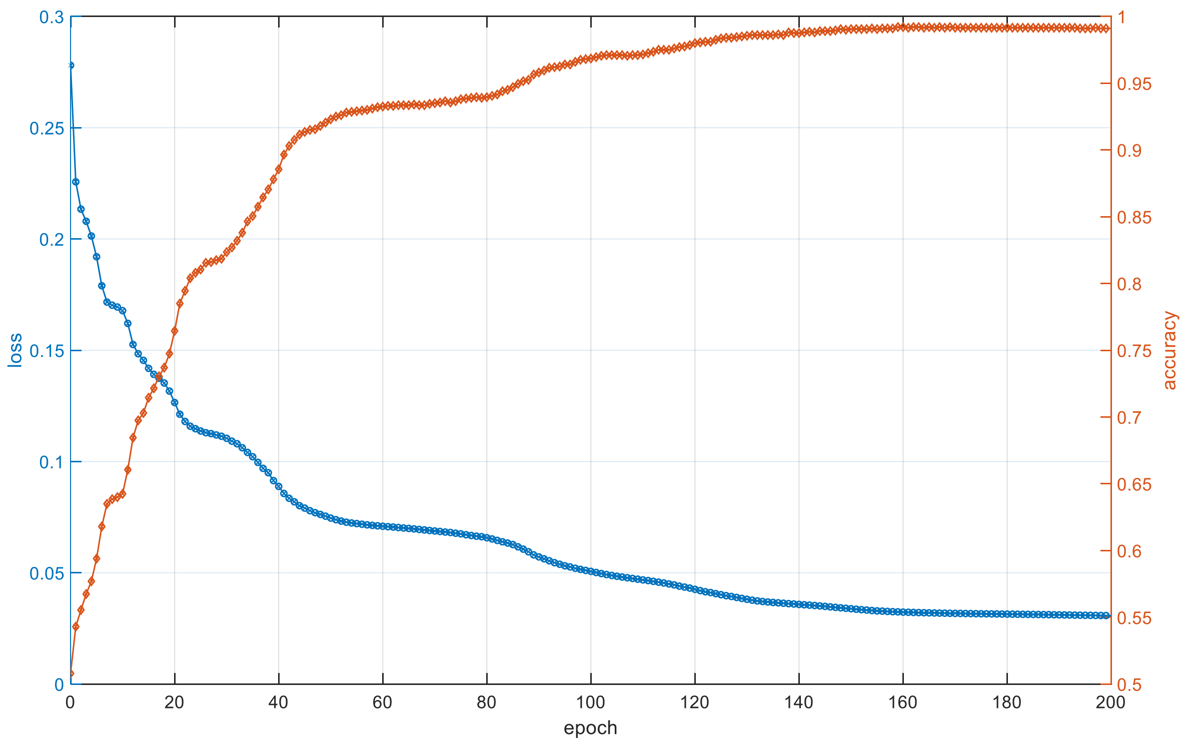

FIG. S4. **Training progress for photonic neural cellular automata.** Cell-wise L_2 loss (blue line) and classification accuracy (orange line) for training PNCA to perform binary image classification of sneakers and trousers classes from fashion-MNIST dataset.

I. INTRODUCTION TO NEURAL CELLULAR AUTOMATA

In this section, we introduce the principles of Neural Cellular Automata (NCA) and how it differs from conventional neural network architectures. Cellular Automata (CA) are computational models composed of a lattice of cells with states that update over time according to simple rules. Crucially, these rules only depend on the local information of neighboring cell states and every cell follows the same local update rule. For example, consider the simple example of a CA known as Elementary Cellular Automata Rule 90. It is a discrete-time CA defined over a 1D lattice of cells with binary states, which we denote by $x_i(t) \in \{0, 1\}$ where $i \in \mathbb{Z}$ is the cell index or lattice site and $t \in \mathbb{N}$ is the discrete time step. The lattice extends infinitely in both directions. Rule 90 follows the simple local update rule: $x_i(t+1) = x_{i-1}(t) \oplus x_{i+1}(t)$, i.e. a cell's next state is given by the logical XOR of its neighboring cell states. This local update rule can also be depicted visually in a truth table as shown in Fig. S5a. The top row shows the local 3-cell neighborhood cell states for all 8 possible cases, and the bottom row shows the updated cell state. This local update

is applied uniformly and simultaneously to all cells in the 1D lattice. The global effect of this local update rule can be visualized using a space-time diagram with the horizontal axis representing cell lattice sites and the vertical axis representing successive time steps. An example is shown in Fig. S5b for an initial condition in which only a single cell is live (has state 1) and all other cells are dead (has state 0). In this case, the space-time diagram is in the shape of a Sierpinski Triangle fractal. A common theme for CA is that even very simple local update rules can lead to global complex or emergent phenomena such as fractals, chaos, synchronization, self-organization, and solitons [1]. It has also been proven that even simple elementary rules are capable of Turing-universal computation [2].

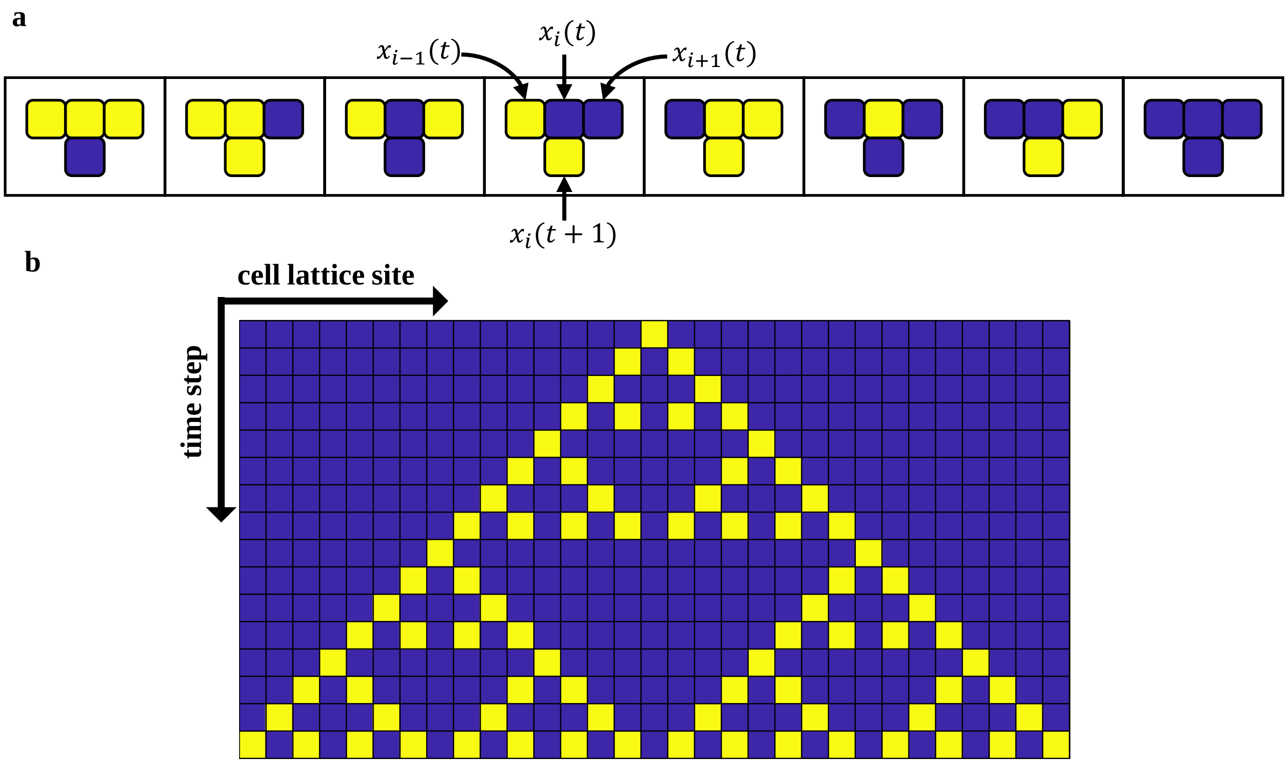

FIG. S5. **Elementary Cellular Automata Rule 90**. (a) Truth table for local update rule. (b) Space-time diagram for Rule 90 starting from a single live cell.

However, although CA are capable of universal computation, it is not always obvious how to harness the complex dynamics in CA to perform *useful* computational tasks. Indeed, much of the early work in CA focused on studying human-designed or bespoke local update rules. In general, we can consider a lattice of cells indexed by lattice site numbers $i \in \mathbb{N}$ with states $\mathbf{x_i} \in \mathbb{C}^d$, where d is the number of channels for each cell (i.e. each cell state is given by a d-dimensional vector). Each cell interacts locally in an m-cell neighborhood $\mathbb{M}_i$ according to a fixed update rule. We

consider discrete-time synchronous updates $t \in \mathbb{N}$ for cells:

$$\mathbf{x_i}(t+1) = f(\mathbf{x_{m_{i1}}}(t), \mathbf{x_{m_{i2}}}(t), \mathbf{x_{m_{i3}}}(t), \cdots) \ , \tag{1}$$

where $m_{i1}, m_{i2}, m_{i3}, \cdots \in \mathbb{M}_i$ are the lattice sites in the local neighborhood of the ith cell and $f : \left(\mathbb{C}^d\right)^m \to \mathbb{C}^d$ is the local update rule.

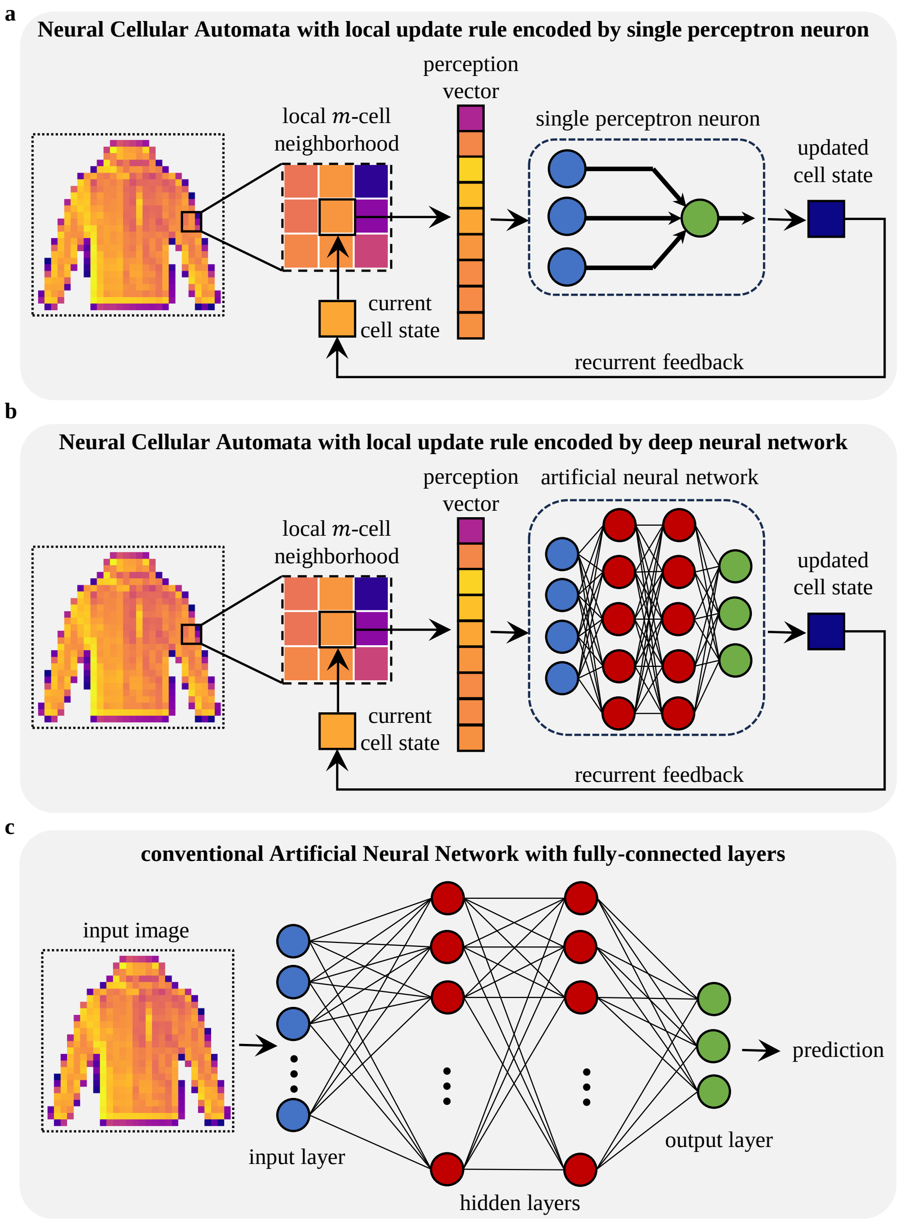

FIG. S6. **Network Topology**. Examples of Neural Cellular Automata (NCA) encoding the local update rule for Cellular Automata (CA) using (a) single perceptron neuron or (b) a deep neural network. (c) Conventional artificial neural networks are feed-forward networks requiring fully-connected layers with global information.

The key innovation of the Neural Cellular Automata (NCA) approach is to use modern deep learning techniques to discover local update rules f that can perform specific tasks [3]. To do this, the cell states are continuous and the local update rule is encoded by a neural network f_θ with parameters θ to be trained. The input to the neural network is a vector composed of the local m-cell neighborhood. For example, Fig. S6a shows the simplest possible example in which the local update rule is encoded by a single perceptron neuron, which is parameterized by its linear input weights. The output of the neuron represents the updated cell state, which undergoes recurrent feedback for the next time step iteration. Therefore, NCA is a special kind of recurrent neural network. We showed in our experimental implementation of NCA using photonics that a local update rule encoded by just a single neuron with 3 parameters can still perform surprisingly complex tasks. Fig. S6b shows the more general case in which the local update rule is encoded by a deeper neural network with more parameters. In order to perform tasks such as image classification, ancillary steps such as alive cell masking and global averaging for label prediction are required (e.g., see Supplementary Section III for more details). The NCA can be trained using the standard backpropagation-through-time algorithm (Fig. S3). It is also common to consider NCA with stochastic updates in which cell states only update with a given probability during each time step. For simplicity, we consider only deterministic NCA in our work. In contrast, the typical network topology of a conventional artificial neural network such as Multi-Layer Perceptron (MLP) is shown in Fig. S6c. It contains an input layer, hidden layer/s, and an output layer arranged in a purely feed-forward network. The layers are fully-connected and require global information. Therefore, NCA can be very parameter-efficient compared to MLPs since the number of parameters needed to encode local update rules is generally far fewer than the number of parameters needed to encode network layers with global information. The process of self-organization through local interactions in NCA for image classification is fundamentally different to the process by which MLPs classify images.

II. EXAMPLES OF HOW TO IMPLEMENT PNCA

In this section, we give more examples of how PNCA may be implemented using existing photonic neural network hardware. We focus on time-multiplexed photonic networks, however, we note that analogous results can be obtained using spatial or wavelength multiplexing [4]. The general architecture for PNCA is shown in Fig. S7a. Each iteration consists of an input optical encoding (e.g., setting initial conditions or alive cell masking using electro-optic modulators) followed by photonic hardware encoding the update rule for cells in a local neighborhood. The key principle of CA is that cells update according to rules that only depend on local information. Therefore, in PNCA the photonic

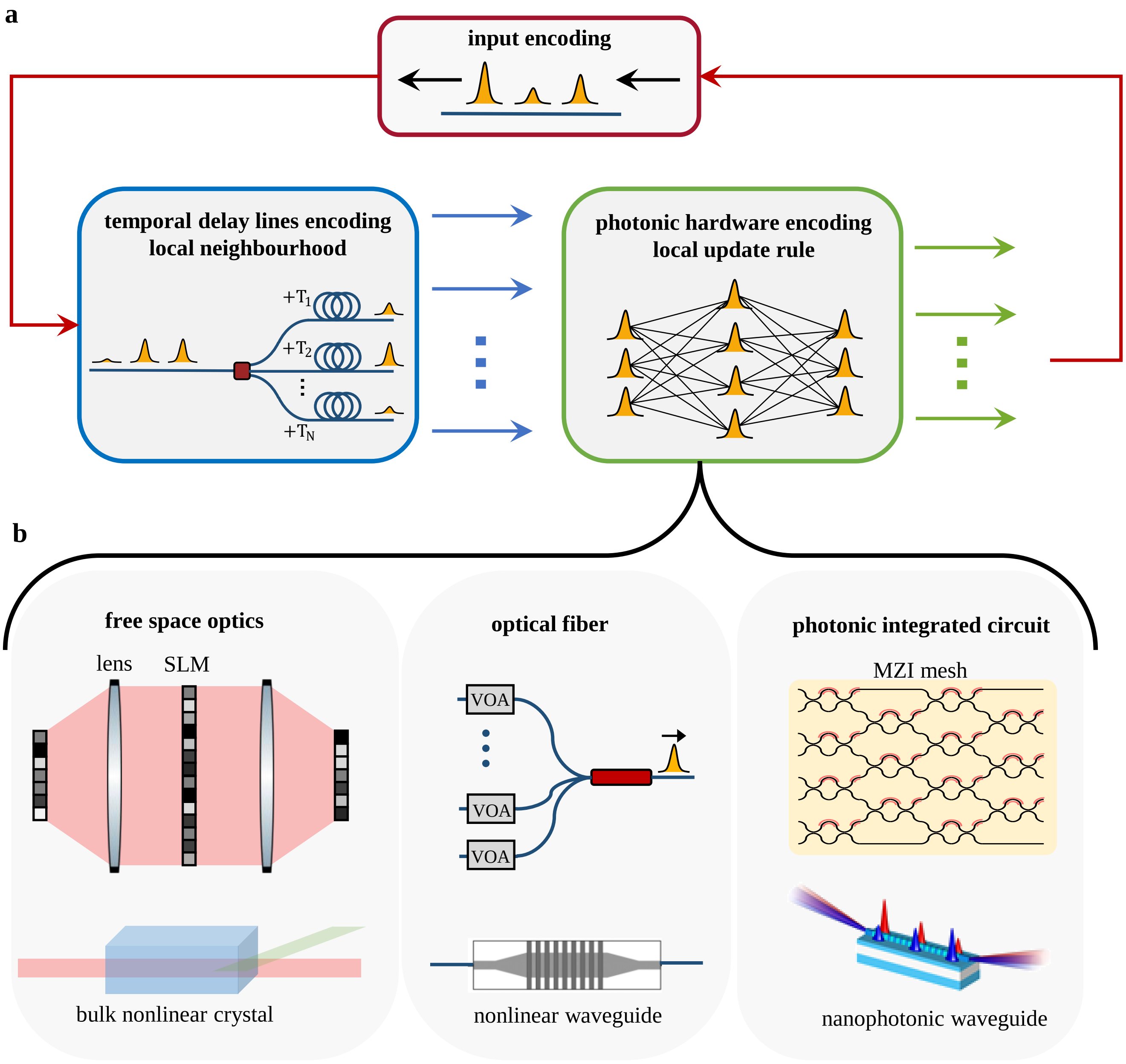

FIG. S7. **PNCA architecture**. (a) The general architecture for PNCA using time-multiplexing includes input encoding of optical cells, temporal delay lines to encode the local cell neighborhood, and photonic hardware to encode the local update rule. (b) The photonic hardware for the local update rule can be implemented using many different methods including free space optics, optical fiber, and photonic integrated circuits.

hardware only encodes for the local update rule, not the global network weights. The local neighbourhood may be encoded, for example, using temporal delay lines to specify which cells will interact in the photonic local update rule. The photonic hardware for the local update rule can be implemented using a myriad of different methods.

Notably, it requires minimal or no changes to existing photonic neural network hardware. For example, PNCA can accommodate any combination of: free space optical neural networks such as coherent [5] or incoherent [6] diffractive neural networks and bulk nonlinear crystals [7]; fiber-based optical networks such as based on time-delay dynamics [8] or wavelength-time interleaving [9], and integrated photonic neural networks such as Mach-Zehnder interferometer meshes [10], cross-bar arrays [11] and nanophotonic nonlinear waveguides [12]. These examples are not an exhaustive list, but rather intended to show how switching to the PNCA architecture can potentially expand the functionality of existing photonic neural network hardware.

III. STEP-BY-STEP EXPLANATION OF DATA PROCESSING PROCEDURE

In this section, we give a detailed overview of the steps used to perform self-organized image classification using the experimentally implemented PNCA. For each input image, we performed the following steps:

1. Pre-processing: prepare the data to be acceptable as input to the PNCA. This step is performed on an external control computer.
    (a) Concatenate the columns of the 28×28 input image to flatten it into a 784×1 vector.
    This is necessary because our experimental implementation of PNCA only accepts a 1D input sequence. However, this step can be skipped if the PNCA can accept 2D imputs.
    (b) Calculate alive cell mask: any pixel with initial value ≥ 0.1 is labelled as *alive* and any pixel with initial value < 0.1 is labelled as *dead*.
2. Neural Cellular Automata: iterate the local update rule for cells. This step is performed in the photonic hardware. For each iteration $t = 1, 2, \cdots, 21$:
    (a) Alive cell masking: cell values represented by the 784×1 vector are modulated onto the amplitude of light pulses. Additionally, the amplitude of any light pulse corresponding to a dead cell is set to 0.
    (b) Update cell values: propagate light pulses through the photonic neural network encoding the local update rule. For example, in this case, it is a single photonic neuron consisting of a multi-arm interferometer and nonlinear PPLN waveguide.
    (c) Feed back cell values: return light pulses back to the start of the network for the next iteration.
    In our experiment, this was done optoelectronically by photodetecting the light pulse after the network

and then reinjecting it electro-optically. However, the feedback can also be performed all-optically using a sufficiently long optical delay line.

3. Prediction: assign the output image classification label. This step is performed on an external control computer.

   (a) Alive cell average: calculate the average cell value of alive cells (i.e. do not include dead cells) after the final iteration.

   Although this step can also be done in photonic hardware, it is not necessary to perform it fast in real-time since it is only computed once after all cell update iterations are completed.

   (b) Output label: the averaged alive cell value gives the probability of the input image belonging to class 1. If the value is closer to zero, then the label is class 0 (e.g. sneaker), otherwise the label is class 1 (e.g. trouser).

   In this experiment, we only performed binary (2-class) classification. However, a more general PNCA classifying more than 2 classes can use the standard softmax activation to assign probabilities and class labels.

IV. OPTOELECTRONIC VS. ALL-OPTICAL FEEDBACK

In this section, we describe possible implementations of the memory in PNCA. Recurrent feedback is needed to input cell states between iterations of the local update rule. The first case is an optoelectronic memory making use of a measurement-feedback scheme. Fig. S8a shows a possible implementation using a time-multiplexed network. Input cell states and alive masking are performed using an amplitude modulator, e.g. an electro-optic modulator, to set the amplitude of light pulses representing cells. Time-delayed copies of the pulses are used to implement the local m-cell neighborhood, which comprises the perception vector that is input to the local update rule. For example, in our experimental setup (see Experimental realization of PNCA), the local update rule is encoded by a single neuron with linear weights set by variable optical attenuators and nonlinear activation performed by a periodically-poled lithium niobate (PPLN) waveguide. Other photonic neural network hardware with more neurons and parameters can also be used to encode the local update rule (e.g., see Supplementary Section II). The updated cell states are photodetected and stored on an electronic memory (e.g., FPGA), which are then electro-optically reinjected for the next iteration of the PNCA. The measurement-feedback scheme is a simple and convenient method for cell state feedback, however, its speed is ultimately limited by the bandwidth of the optoelectronic conversions. The second case overcomes this

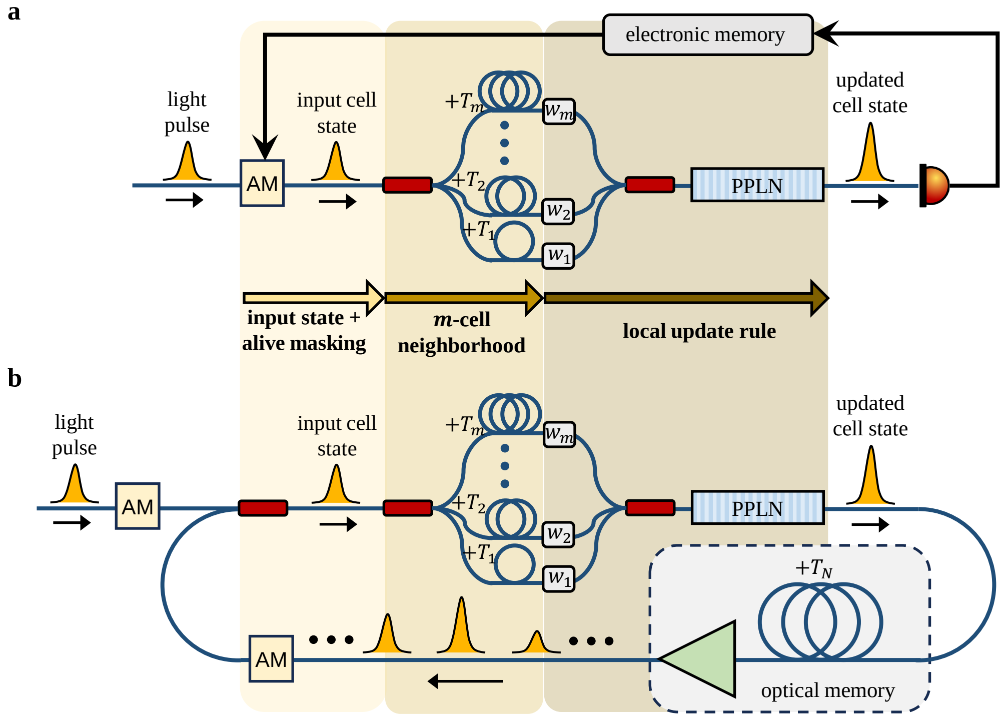

FIG. S8. **Photonic Neural Cellular Automata Memory.** Example of an implementation using: (a) optoelectronic conversion, electronic memory and amplitude modulator (AM); and (b) all-optical memory and AM.

issue by using an all-optical memory. Fig. S8b shows a possible implementation using a time-multiplexed network. Input cell states and alive masking are performed using an amplitude modulator (AM) to set the initial amplitude of light pulses representing cells. This initial AM is used to set the initial conditions (i.e. image pixel values) and is not active beyond the initial $t = 0$ iteration. The PNCA computation based on local m-cell neighborhood and local update rule proceeds similarly as before. The updated cell state pulse passes through an optical memory comprised of an optical delay line with time delay T_N, where N is the number of cells in the network, and an optical amplifier (e.g., erbium-doped fiber amplifier or optical parametric amplifier) to compensate the optical loss. A second AM is needed in this case to perform the alive masking between each iteration in the optical feedback line, which inputs the pulses for the next iteration. Note that the AMs can also be implemented using previously demonstrated all-optical switches based on ultrafast nonlinear optics [13] so that it is not limited by electronic bandwidths. Therefore, the entire PNCA can be implemented all-optically without any electronic bottleneck. This all-optical method requires a long delay line with delay $+T_N$ to ensure that cells in each iteration do not undesirably interfere with cells in subsequent iterations. The network size N is only limited by the length of available optical fiber.

V. COMPARISON TO OTHER OPTICAL NEURAL NETWORKS

Architecture	Image Dataset (Image Size)	Input Update Method	Input Updates per Image	Input Update Rate	Weight Update Method	Weight Updates per Image	Weight Update Rate	Linear Operation Method	Nonlinear Activation Method	Image Inference Time	Claimed Energy Efficiency	Claimed Operation Speed
PNCA (this work)	fashion-MNIST (28×28)	Electro-optic modulator	15680 [a]	12 GHz	Variable optical attenuators	0	0	Optical	Optical	**1.307 μs**	5.6 TOPs/J	84 GOPs/s
CNN [9]	MNIST (30×30)	Electro-optic modulator	900	11.9 GHz	Spectral waveshaper	1 [b]	2 Hz	Optical	Digital Electronic	>500 ms [*]	N/A	11.321 TOPs/s
D^2NN [5]	MNIST (28×28)	Digital micro-mirror display	3	14.989 kHz	Spatial light modulator	3	422.4 Hz	Optical	Opto-electronic	20.7 ms [c]	0.717 TOPs/J	**114.1 TOPs/s**
VCSEL-ONN [14]	MNIST (28×28)	VCSEL	15900 [d]	1 GHz	VCSEL array	15900 [d]	1 GHz	Opto-electronic	Opto-electronic	15.9 μs	**140 TOPs/J**	~1 TOPs/s [e]
PNN [7]	MNIST (14×14)	Digital micro-mirror display	8 [f]	200 Hz	Digital micro-mirror display	8 [f]	200 Hz	Digital Electronic	Optical	>40 ms [*]	N/A	N/A
CNN [15]	fashion-MNIST (14×14)	Variable optical attenuators	169	1 kHz	Electrical PCM cell	4	1.193 MHz [g]	Optical + Digital Electronic	Digital Electronic	0.17 s	5 TMACs/J	25 Gb/s
CNN [11]	MNIST (28×28)	Electro-optic modulator	784	2 GHz	Optical PCM cell	0	0	Optical + Digital Electronic	Digital Electronic	>8.1 μs [*]	58.8 TMACs/J	2 TMACs/s
OONA [6]	QuickDraw (28×28)	Image intensifier	1	10 Hz	Spatial light modulator	0	0	Optical + Digital Electronic	Opto-electronic + Digital Electronic	>5 ms [*]	N/A	N/A

TABLE I. **Comparison of state-of-the-art optical neural networks performing image classification**.

[a] Assuming $t = 20$ iterations ($784 \times 20 = 15680$)

[b] Assuming the waveshaper was only updated once in between the convolutional and fully-connected layers

[c] Assuming a 3-layer D^2NN with DPU working cycle of 6.9 ms including sensor exposure time

[d] Assuming a $784 \rightarrow 100 \rightarrow 10 \rightarrow 10$ 3-layer model with arbitrary waveform generator using 5 VCSELs per acquisition ($784 \times 20 + 100 \times 2 + 10 \times 2 = 15900$)

[e] Assuming a compute density of 6 TOPs/mm^2/s and a VCSEL chip area of $25 \times 80\mu\text{m}\times 80\mu\text{m}$

[f] Assuming 4 independent channels with 2 SHG transformation steps per channel

[g] Assuming 282 ns write time and 556 erase time per GST cell switching cycle

[*] digital electronic processing time not reported

In this section, we compare the experimentally demonstrated PNCA to other state-of-the-art optical neural networks

performing image classification. We consider system-level demonstrations of images with size at least 14×14 since anything smaller is not representative. We compare only the best value for experimentally reported hardware and not theoretically proposed or future devices. We consider the image inference time, which is the minimum time needed to classify one image, to be a key figure of merit for photonic hardware accelerators. This can be calculated from considering the number of input updates and weight updates needed for each image. For example, in the case of our PNCA, the number of input updates required per image (assuming $t = 20$ iterations of the local update rule) is $784 \times 20 = 15680$. The input update rate is limited by the bandwidth of our electro-optic modulator, which is 12 GHz. One of the crucial points about PNCA is that there is no weight updates required, so this does not contribute to the inference time. We ignore the light propagation time since it is almost always negligible compared to the time taken for input/weight updates. Therefore, the image inference time is 15680/12 GHz = 1.307μs. Another key consideration is how the linear/nonlinear operations are implemented since using electronics for parts of the computation will add significant time and overhead, which is usually not reported. The experimental PNCA implements both linear and nonlinear operations in the optical domain so there is minimal overhead. Besides the equipment control electronics, optoelectronics was used in storing and reinjecting the cell states between iterations, but this does not affect the speed of the computation since the time needed to reinject a single cell state is much less than the time taken for a complete iteration. Similarly, the time taken to output classification labels electronically in the last step is much less than the time taken to complete all iterations. During each iteration, each cell experiences 1 alive masking operation, 3 MAC operations, and 1 nonlinear activation, hence giving a total of 7 operations per cell (assuming 1 MAC = 2 OPs). Therefore, the current operation speed is 7 OPs $\times$12 GHz = 84 GOPs/s. The average optical power was 15 mW, so we can estimate the optical energy consumption for the computation as (84 GOPs/s)/15 mW = 5.6 TOPs/J. However, we note that the power consumption of the control electronics is much higher than the optical power, which means the overall wall-plug energy efficiency is likely much worse than implied by just the optical energy consumed during the computation. Interestingly, we see that a higher claimed operation speed does not directly translate into a faster inference time. The claimed operation speeds often assume full utilization of the photonic hardware, which ignores important system-level constraints such as input and/or weight updates that can drastically slow the system when performing a useful image classification task. Our PNCA experimental implementation is the only end-to-end photonic neural network combining linear optical operations, nonlinear optical operations, fast input update speeds, static weights, and no electronic bottleneck.

VI. $\chi^{(2)}$ NONLINEAR OPTICAL ACTIVATION FUNCTIONS

In this section, we describe the equations governing the pump-depleted second harmonic generation (SHG) in the periodically-poled lithium niobate (PPLN) waveguide used for nonlinear activations in the main text experiments. SHG arises due to the second-order ($\chi^{(2)}$) parametric processes in non-centrosymmetric crystals such as lithium niobate. This $\chi^{(2)}$ nonlinearity is of the ultrafast variety with an effectively instantaneous response time, which is only limited in practice by the phase-matching bandwidth of the device. Unlike other slower optical nonlinearities [16, 17] that are incoherent, the PPLN waveguide can correctly handle both positive and negative valued input light amplitudes and is a completely travelling-wave process (no resonator required). In its simplest form, the SHG process is governed by the nonlinear coupled-mode equations [18] in Eq. 2:

$$\frac{d}{dz}A(z) = -i\kappa^* A^*(z)B(z)\exp(-i2\Delta z) \ , \tag{2a}$$

$$\frac{d}{dz}B(z) = -i\kappa\left[A(z)\right]^2\exp(i2\Delta z) \ , \tag{2b}$$

where $A(z)$ is the amplitude of the fundamental harmonic light of frequency ω, $B(z)$ is the amplitude of the second harmonic light of frequency 2ω, κ is the nonlinear coupling coefficient, Δ is the phase-mismatch parameter between the ω and 2ω light waves, and z is the propagation distance along the waveguide's longitudinal direction. In the case of our experiments, the input value is encoded onto the coherent light amplitude of the fundamental harmonic wave and we restrict our attention to only real-valued inputs. Solving the initial value problem Eq. 2 with $A(0) = A_0$ and $B(0) = 0$ for a waveguide of length L gives the nonlinear activation function, $g : \mathbb{R} \to \mathbb{R}$, as the output fundamental harmonic light amplitude $A(L) = g(A_0)$. Suppose that $[A^+(z),\ B^+(z)]$ with $A^+(0) = A_0$ and $B^+(0) = 0$ is a solution to Eq. 2 with initial values $A^+(0) = A_0$ and $B^+(0) = 0$. Then, by direct substitution, we see that $[A^-(z) = -A^+(z),\ B^-(z) = B^+(z)]$ is also a solution with initial values $A^-(0) = -A_0$ and $B^-(0) = 0$. Therefore, the nonlinear activation function $g(\cdot)$ is an anti-symmetric (odd) function, i.e. $g(-x) = -g(x)$ for $x \in \mathbb{R}$. For example, in the simplified case of continuous-wave light and perfect phase-matching ($\Delta = 0$), the exact analytical solution to the initial value problem Eq. 2 with $A(0) = A_0$ and $B(0) = 0$ is:

$$A(z) = A_0\text{sech}(\kappa A_0 z),\ B(z) = -iA_0\text{tanh}(\kappa A_0 z) \ . \tag{3}$$

Clearly, the nonlinear activation function $A(L) = g(A_0)$ is anti-symmetric (odd) for this simple case. Alternatively, we could also use the second harmonic output $B(L) = f(A_0)$ if we desire a symmetric (even) nonlinear activation

function, however, the output would be at a different frequency to the input. The case for short laser pulses is more complicated and can only be solved numerically, e.g. see Ref. [12].

VII. SIGNAL-TO-NOISE RATIO

In this section, we elaborate on the signal-to-noise (SNR) of the PNCA and the maximum noise tolerance. A key point for PNCA is that the information (e.g. image class label) is encoded in the global or collective cell states, and not necessarily reliant on any individual cell. Therefore, we can assign the alive cell average as the relevant "signal" and track its evolution across iterations for a specific image class. The noise can then be considered as the standard deviation of the alive cell average distribution. For example, Fig. S9(a) shows the SNR at different iterations of the experimentally demonstrated PNCA (see main text Fig. 4) for fashionMNIST trouser images. Initially, the SNR is low since the alive cell states are the same as the input image pixel values and have a large variance. Interestingly, the SNR increases during the first few iterations before decreasing and eventually plateauing. This is because the most rapid changes due to the self-organizing behaviour occur in the first few iterations.

We can also study how the noise from specific optical operations impacts the classification accuracy. In our experimental implementation of PNCA, we carefully characterized the noise (see main text Fig. 3), which can be

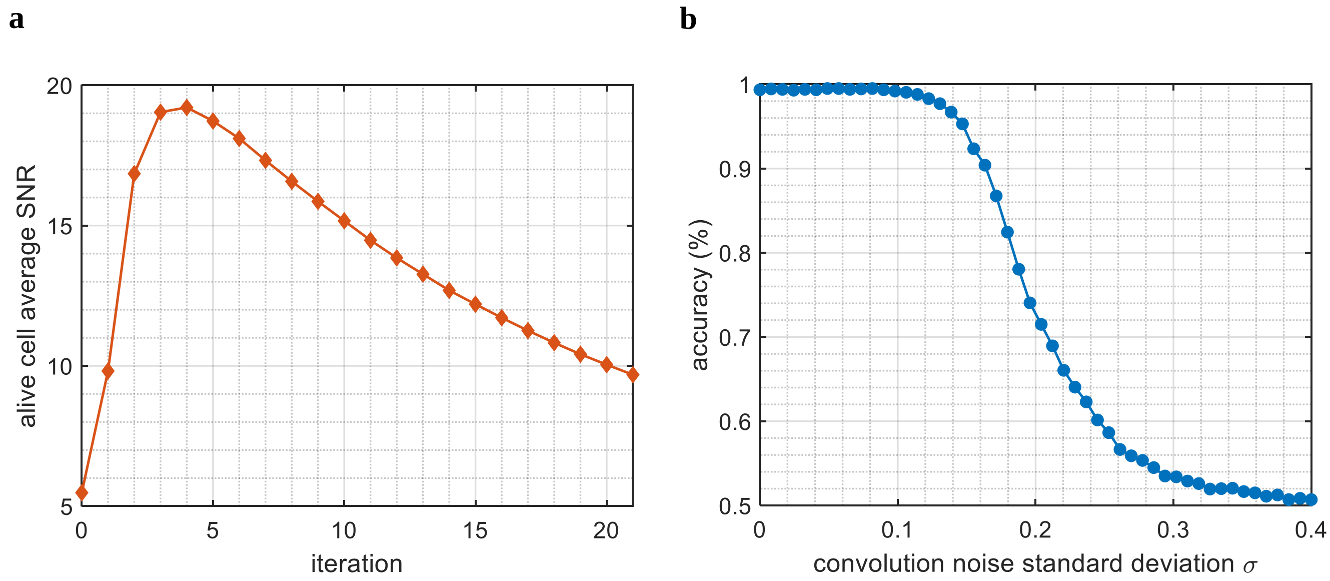

FIG. S9. **Noise Properties of PNCA** (a) Signal-to-noise ratio at each iteration of the experimentally demonstrated PNCA tested on fashionMNIST trouser images. (b) Simulated PNCA accuracy for binary image classification of fashionMNIST sneaker and trouser images when the convolution noise standard deviation is varied.

considered typical of photonic hardware. We showed that PNCA was robust to this level of noise. To determine the maximum degree of noise robustness, we now simulate the PNCA classification accuracy for the same task of binary image classification of fashionMNIST sneaker and trouser images as the noise is increased. For simplicity, we vary only the standard deviation σ of the noise of the linear operations (convolutions) since it is the dominant noise source, and keep the input and nonlinear activation noise fixed (as shown in main text Fig. 3). The accuracy for different levels of noise σ is shown in Fig 9(b). The PNCA maintains near-perfect binary classification accuracy up to $\sigma \approx 0.1$, which is > 3 times the actual experimental noise level of $\sigma = 0.0327$. The PNCA maintains $> 90\%$ accuracy up to > 5 times the experimental noise level. The PNCA doesn't completely fail until $\sigma \approx 0.35$, which is > 10 times the experimental noise level. This emphasizes the fact that the noise properties of specific operations acting on individual cells are not straightforwardly correlated with the emergent global properties of the alive cell averages.

[1] S. Wolfram, Statistical mechanics of cellular automata, Reviews of modern physics **55**, 601 (1983).

[2] M. Cook *et al.*, Universality in elementary cellular automata, Complex systems **15**, 1 (2004).

[3] E. Randazzo, A. Mordvintsev, E. Niklasson, M. Levin, and S. Greydanus, Self-classifying mnist digits, Distill **5**, e00027 (2020).

[4] L. Yuan, Q. Lin, M. Xiao, and S. Fan, Synthetic dimension in photonics, Optica **5**, 1396 (2018).

[5] T. Zhou, X. Lin, J. Wu, Y. Chen, H. Xie, Y. Li, J. Fan, H. Wu, L. Fang, and Q. Dai, Large-scale neuromorphic optoelectronic computing with a reconfigurable diffractive processing unit, Nature Photonics **15**, 367 (2021).

[6] T. Wang, M. M. Sohoni, L. G. Wright, M. M. Stein, S.-Y. Ma, T. Onodera, M. G. Anderson, and P. L. McMahon, Image sensing with multilayer nonlinear optical neural networks, Nature Photonics **17**, 408 (2023).

[7] L. G. Wright, T. Onodera, M. M. Stein, T. Wang, D. T. Schachter, Z. Hu, and P. L. McMahon, Deep physical neural networks trained with backpropagation, Nature **601**, 549 (2022).

[8] G. H. Li, C. R. Leefmans, J. Williams, and A. Marandi, Photonic elementary cellular automata for simulation of complex phenomena, Light: Science & Applications **12**, 132 (2023).

[9] X. Xu, M. Tan, B. Corcoran, J. Wu, A. Boes, T. G. Nguyen, S. T. Chu, B. E. Little, D. G. Hicks, R. Morandotti, *et al.*, 11 tops photonic convolutional accelerator for optical neural networks, Nature **589**, 44 (2021).

[10] Y. Shen, N. C. Harris, S. Skirlo, M. Prabhu, T. Baehr-Jones, M. Hochberg, X. Sun, S. Zhao, H. Larochelle, D. Englund, *et al.*, Deep learning with coherent nanophotonic circuits, Nature Photonics **11**, 441 (2017).

[11] J. Feldmann, N. Youngblood, M. Karpov, H. Gehring, X. Li, M. Stappers, M. Le Gallo, X. Fu, A. Lukashchuk, A. S. Raja, *et al.*, Parallel convolutional processing using an integrated photonic tensor core, Nature **589**, 52 (2021).

[12] G. H. Y. Li, R. Sekine, R. Nehra, R. M. Gray, L. Ledezma, Q. Guo, and A. Marandi, All-optical ultrafast relu function for energy-efficient nanophotonic deep learning, Nanophotonics **12**, 847 (2022).

[13] Q. Guo, R. Sekine, L. Ledezma, R. Nehra, D. J. Dean, A. Roy, R. M. Gray, S. Jahani, and A. Marandi, Femtojoule femtosecond all-optical switching in lithium niobate nanophotonics, Nature Photonics **16**, 625 (2022).

[14] Z. Chen, A. Sludds, R. Davis III, I. Christen, L. Bernstein, L. Ateshian, T. Heuser, N. Heermeier, J. A. Lott, S. Reitzenstein, *et al.*, Deep learning with coherent vcsel neural networks, Nature Photonics **17**, 723 (2023).

[15] W. Zhou, B. Dong, N. Farmakidis, X. Li, N. Youngblood, K. Huang, Y. He, C. David Wright, W. H. Pernice, and H. Bhaskaran, In-memory photonic dot-product engine with electrically programmable weight banks, Nature Communications **14**, 2887 (2023).

[16] J. Feldmann, N. Youngblood, C. D. Wright, H. Bhaskaran, and W. H. Pernice, All-optical spiking neurosynaptic networks with self-learning capabilities, Nature **569**, 208 (2019).

[17] F. Ashtiani, A. J. Geers, and F. Aflatouni, An on-chip photonic deep neural network for image classification, Nature **606**, 501 (2022).

[18] T. Suhara and M. Fujimura, *Waveguide nonlinear-optic devices*, Vol. 11 (Springer Science & Business Media, 2013).
